# RhoA/C inhibits proliferation by inducing the synthesis of GPRC5A

**DOI:** 10.1038/s41598-020-69481-2

**Published:** 2020-07-27

**Authors:** Lukas Richter, Viktoria Oberländer, Gudula Schmidt

**Affiliations:** Institute for Experimental and Clinical Pharmacology and Toxicology, Albert-Ludwigs-University of Freiburg, Albert-Str. 25, 79104 Freiburg, Germany

**Keywords:** Breast cancer, RHO signalling, Breast cancer

## Abstract

Rho GTPases are important regulators of many cellular functions like cell migration, adhesion and polarity. The molecular switches are often dysregulated in cancer. We detected Rho-dependent upregulation of the orphan seven-transmembrane receptor G-protein-coupled receptor family C group 5 member A (GPRC5A). GPRC5A is highly expressed in breast cancer whereas in lung cancer, it is often downregulated. Here, we analyzed the function of GPRC5A in breast epithelial and breast cancer cells. Activation or expression of RhoA/C led to GPRC5A-dependent inhibition of proliferation and reduction of the colony forming capacity of benign breast epithelial cells. This effect is based on an inhibition of EGFR signalling. Knockout of retinoic acid induced 3 (RAI3, the gene for GPRC5A) in breast cancer cells increased cell division, whereas Rho activation had no effect on proliferation. Knockout of RAI3 in benign breast epithelial cells led to decrease of EGFR expression and diminished proliferation.

## Introduction

Rho GTPases are molecular switches regulating important cellular functions like gene transcription and proliferation^1^. They are well known regulators of the cytoskeleton and are essentially involved in cell migration, adhesion and polarity. Recent studies showed that dysregulation of Rho GTPases plays a pivotal role in cancer development^2^ regulating proliferation, invasion and metastasis of various types of tumor cells^3–5^. In epithelia, progression from a persistent to an invasive phenotype requires loss of epithelial polarity and of cellular adhesion. This epithelial-to-mesenchymal transition (EMT) includes a change in gene expression pattern induced by several transcription factors, like Snail, ZEB1 or Twist^6, 7^. Recently, we showed that pro-migratory genes like PTGS2 and serpine1 are upregulated in a RhoA/C specific manner^8^. Moreover, RhoC-dependent expression of Cox2 was involved in migration and invasion. In our studies, we noticed that expression or activation of Rho GTPases dramatically inhibited proliferation of MCF10A breast epithelial cells. Activation of Rho GTPases led to upregulation of the G-protein-coupled receptor family C group 5 member A (GPRC5A). GPRC5A is an orphan seven-transmembrane receptor identified in 1998 to be encoded by the retinoic acid (RA)-induced gene 3 (RAI3)^9^. RAI3 is dysregulated in several human cancer entities. Interestingly, in tissues with high GPRC5A expression (lung), malignant cells are associated with reduced expression, indicating a tumor-suppressive role of the membrane protein. Studies with GPRC5A knockout mice suggested a tumor-suppressive function of the protein in lung adenocarcinoma. It was shown that GPRC5A interacts with the epidermal growth factor receptor (EGFR) thereby preventing its signaling and reducing proliferation of lung cancer cells^10^. Consistently, RAI3 was downregulated in more than 60% of lung tumors^11^. In sharp contrast, GPRC5A is highly expressed in breast cancer, colorectal and pancreatic carcinoma while its expression is low in the respective healthy tissues (for review see^12^). In line with the contrasting expression, recent analysis of GPRC5A function revealed a controversial role in different cancer entities: Expression of GPRC5A in non-tumorigenic pancreatic epithelial cells promoted colony formation^13^. Consistently, knockdown of RAI3 in pancreatic cancer cells led to decreased proliferation and reduced migration, indicating a pro-metastatic role for GPRC5A in pancreatic cancer^14^. In colorectal cancer, elevated GPRC5A expression is associated with worse prognosis and induces cell proliferation and tumorigenesis in a colitis-associated cancer model^15^. A tumor-suppressive effect of GPRC5A has been shown in MDA-MB-231 breast cancer cells^16^. In these cells knockdown of RAI3 induced proliferation, migration and invasion. In contrast, silencing of GPRC5A had no effect on MCF7 cells with low EGFR levels, indicating a direct effect of GPRC5A on the EGFR stability and/or EGF-induced proliferation^16^.

We intended to analyze the connection between Rho GTPases, GPRC5A expression and proliferation in breast epithelial and cancer cells. In our studies we used the benign breast epithelial cell line MCF10A with inducible expression of Rho proteins. Moreover, we treated the cells with the bacterial toxins Cytotoxic Necrotizing Factor 1 or Y (CNF1 or CNFY) to activate the endogenous pool of Rho GTPases. The toxins are taken up into mammalian cells by receptor-mediated endocytosis and are released from the endosome into the cytoplasm^17^. Rho proteins are constitutively activated by the bacterial protein toxins which catalyze the deamidation of a specific glutamine residue in Rho proteins and thereby lead to constitutive activation of the GTPases (for review, see^18^). Moreover, we knocked out RAI3 in MDA-MB-231 breast cancer cells and in benign MCF10A breast epithelial cells to study the effects of G-protein receptor deficiency in the absence and presence of Rho activation.

## Materials and methods

### Cell culture and reagents

MCF10A wild-type cell line was purchased from ATCC. MCF-10Atet cells allowing inducible expression of RhoA or RhoC together with GFP under the control of a second generation Tet-regulated transcriptional trans-activator and silencer were generated via nucleofection and have been described previously^8^. All cells were grown in DMEM/F12 medium containing 5% horse serum, 100 U/ml penicillin, 100 µg/ml streptomycin, 20 ng/ml epidermal growth factor, 0.5 µg/ml hydrocortisone, 100 ng/ml cholera toxin and 10 µg/ml insulin. MDA-MB-231 culture medium contains DMDM/F12, 10% FCS, 100 U/ml penicillin and 100 µg/ml streptomycin. The cells were incubated at 37 °C and 5% CO_2_. For the induction of the transgenic overexpression of RhoA and RhoC doxycycline was used at 2 µg/ml. Staurosporine was dissolved in DMSO. Purification of CNF toxins was performed as described previously^19^ and were used at 1 nM. Every 2–3 days, all inhibitors, inducers and toxins were re-added with new medium.

### Colony formation assay

To check for the colony formation capacity cells were seeded in a 6-well plate (500 cells per well). Following overnight attachment, doxycycline was added where indicated and cultured for at least 6 days at 37 °C and 5% CO_2_. The medium was exchanged every three days. The colonies were fixed with glutaraldehyde (6,0% (v/v)) and stained with crystal violet (0,5% (w/v)) for 30 min. Afterwards the fixation staining solution was removed, the colonies were washed carefully with distilled water and dried at room temperature.

### BrdU proliferation assay

As an indicator for proliferation DNA synthesis was measured using the chemiluminescent Cell Proliferation ELISA Kit (Roche) for quantifying the incorporation of 5-bromo-2′-deoxyuridine (BrdU). The cells were seeded in a black flat bottom 96-well plate (5,000 cells per well), allowed to adhere overnight and incubated for 48 h with doxycycline where indicated. Then BrdU was added for 4 h (final concentration 10 µM) and the assay was performed according to the manufacturers protocol. The chemiluminescent signal was detected using a 96-well plate reader (Tecan infinite M200, Tecan Trading AG). Each assay was performed in technical triplicates. Percentage of BrdU incorporation was calculated with the following equation: % BrdU incorporation = (experimental signal-background signal)/(control signal-background signal) × 100.

### Cell viability assay

Metabolic activity was detected measuring the cellular capacity to reduce the indicator dye resazurin to resafurin with the CellTiter-Blue^®^ Cell Viability Assay Kit (Promega). The cells were seeded in a black flat bottom 96-well plate (5,000 cells per well), allowed to adhere overnight and incubated for 48 h with doxycycline, where indicated. After incubation the CellTiter-Blue^®^ Reagent was added for 3 h and fluorescence was detected using a 96-well plate reader (Tecan infinite M200, Tecan Trading AG). Each assay was performed in technical triplicates. Percentage of viable cells was calculated with the following equation: % viable cells = (experimental absorbance-background absorbance)/(control absorbance-background absorbance) × 100.

### qRT-PCR

RNA was isolated from 2D cultures at indicated time points using the RNeasy Mini Kit (Qiagen) according to the manufactures protocol. Total RNA was eluted in RNAse-free distilled H_2_0 and the final concentration was determined on a photometer at 260 nm. For each sample, 1 µg RNA was applicated for cDNA synthesis using QuantiTect Reverse Transcription Kit (Qiagen) according to the manufacturers instructions. Finally, cDNA was diluted 1:10 and amplified using the GoTaq^®^ qPCR Master-Mix (Promega) on a Mastercycler^®^ Realplex (Eppendorf). Raw data were analyzed with LinRegPCR 2012. S29 served as a housekeeping gene reference. (Primers: GPRC5A forward: 5′-GCACTAGGGTCCAGAATGG-3′, GPRC5A reverse: 5′-ACCGTTTCTAGGACGATGC-3′, S29 forward: 5′-GGTTCTCGCTCTTGTCGTGTC-3′, S29 reverse: 5′-ATATCCTTCGCGTACTGACGG-3′).

### Western blot analysis

Western Blot analysis were performed using standard techniques. After removing the medium, the cells were washed once with PBS and then lysed in NP-40 lysis buffer (50 mM Tris–HCL (pH 8.0), 150 mM NaCl and 1% NP-40) containing protease inhibitor (Complete, Roche) and phosphatase inhibitor (Sigma-Aldrich) if necessary. The samples were separated with a 12.5% or 7% SDS-PAGE and blotted using the wet blot method (25 mM Tris–HCl, 192 mM glycine, 20% (v/v) methanol, 100 V, 75 min). Incubation with the primary antibodies were performed overnight. Used antibodies are: anti-RhoA (67B9, Cell Signaling Technologies), anti-RhoC (D40E, Cell Signaling Technologies), anti-GAPDH (6C5, EMD-Millipore), anti-tubulin (DM1A, Santa Cruz), anti-GPRC5A (HPA007928, Atlas Antibodies), anti-EGFR (D38B1, Cell Signaling Technologies), anti-P-EGFR (Y1068, 1H12, Cell Signaling Technologies) and a suitable secondary antibody coupled to horseradish peroxidase (HRP).

### Viral transduction

For virus production, HEK phoenix cells were transfected with pMIBerry empty vector or with pMiBerry containing the RAI3 gene and stimulated with 5 mM sodium butyrate overnight. Transfection was controlled using fluorescent microscopy. Virus containing supernatant was used directly or stored at 4 °C. MCF10A cells were treated with virus containing supernatant (1 ml + 9 ml fresh culture medium) four times for 1 day each. Sufficient transduction was analyzed by red fluorescence before cells were serum starved.

### CRISPR-Cas9 mediated GPRC5A knockout

We performed a knockout of GPRC5A in MDA-MB-231 and MCF10A cells using the CRISPR-Cas9 system. We followed the protocol from Ref.^1^.

To design the targeting components and determine the 20-nt guide sequence (5ʹ GTCCCTGATGGTTGCCGCAA 3ʹ) within the sgRNA including a 5′-NGG PAM (5ʹ TGG 3ʹ), we used the online CRISPR-Cas9 Design tool provided by https://tools.genome-engineering.org. We selected a target site within Exon 2 of the human GPRC5A gene.

For construction of an expression plasmid for sgRNA and Cas9 we used the pSpCas9(BB)-2A-Puro (PX459) V2.0 Vector (AddGene Plasmid #62988). For co-expression of sgRNA and Cas9, the partially complementary oligonucleotides encoding the 20-nt guide sequences were phosphorylated, annealed and ligated into the plasmid. The plasmid was then transformed into competent *E. coli* strain. To verify the sequence of the plasmid we isolated the plasmid DNA from several bacterial cultures and performed sequencing from the U6 promoter.

### Transfection of MDA-MB-231 and MCF10A cells

To perform the knockout, MDA-MB-231 and MCF10A cells were transfected with the sequence verified plasmid. For the Insertion of DNA in mammalian cells Lipofectamin 2000 (Invitrogen, Thermo Fisher Scientific) was used according to the manufacturer's instructions.

For transfection 1 million cells per well were seeded into a 10 cm dish. The confluency was 60–80%. Due to the selectable marker on the pSpCas9(BB)-2A-Puro (PX459) V2.0 Vector the cells were selected through a Puromycin treatment with 1,0 µg/ml Puromycin over two days.

### Clonal isolation of cell lines

After transfection and selection, isolation of clonal cell lines was achieved by serial dilution. After an expansion period the new single cell lines where each tested for a GPRC5A knockout through PCR and Western Blot.

### Statistical analysis

For all statistical analysis GraphPad Prism 5.0 was used. All values, bars and error bars represent mean + standard deviation (SD). A p-value of < 0.05 was considered as statistically significant.

## Results

### RhoA/C expression or activation inhibits proliferation of MCF10A human breast epithelial cells

**Figure 1:**
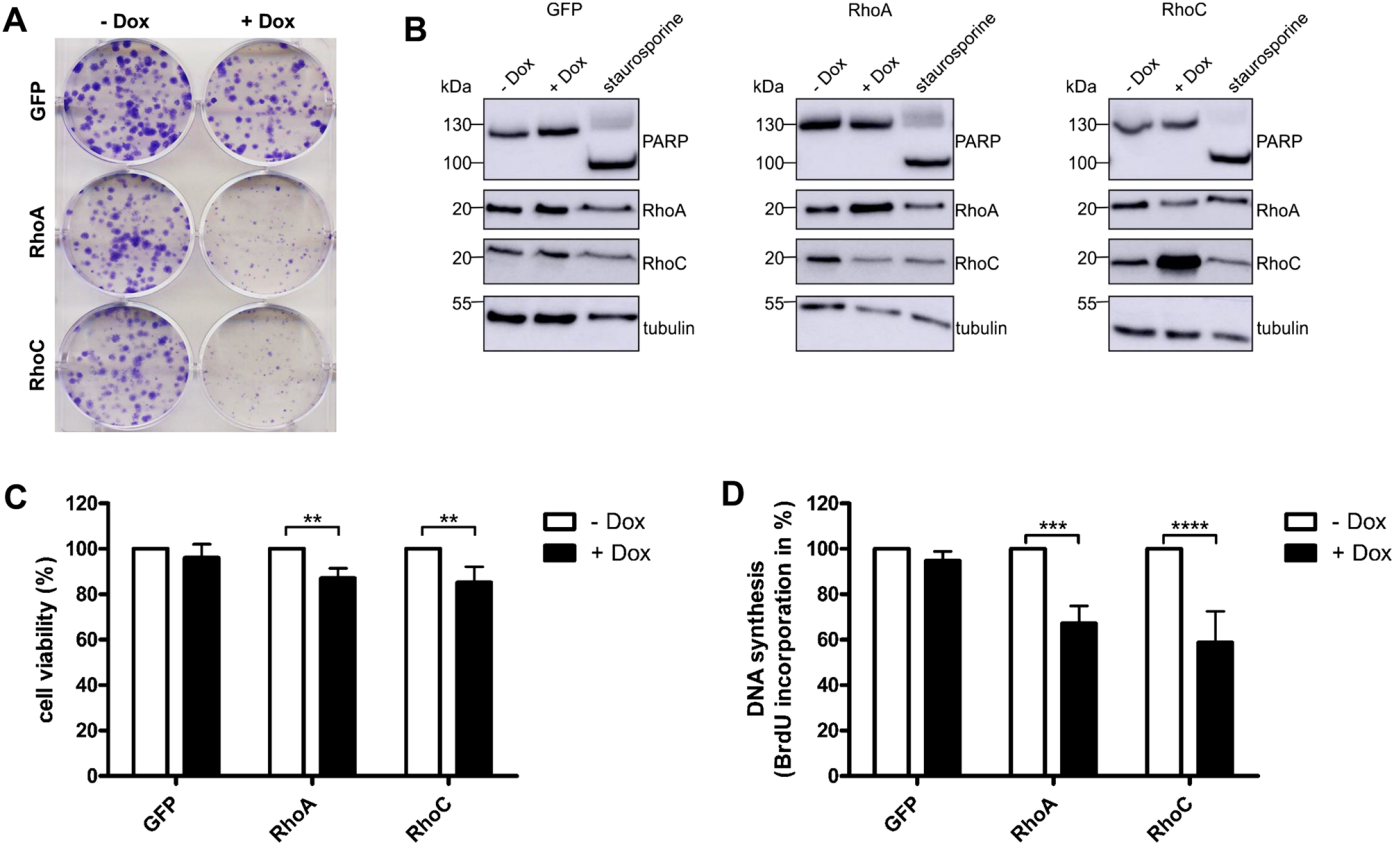
Expression of RhoA or RhoC inhibits proliferation of MCF10A cells. MCF-10Atet cells were transfected with RhoA plus GFP (RhoA), RhoC plus GFP (RhoC) or GFP (GFP, empty vector control)-containing constructs under the control of a tet-ON-promoter. Expression of GFP/Rho was induced by addition of 2 µg/ml doxycycline. (**A**) Crystal violet-stained colonies of GFP-, RhoA- or RhoC-expressing cells after 6 days treatment with (+ Dox) or without (−Dox) doxycycline (n = 3). (**B**) Analysis of apoptosis induction after expression of RhoA, RhoC or GFP for 48 h, respectively. PARP cleavage was detected by Western Blot analysis. Treatment of the cells with 1 µM staurosporine for 16 h was used as a positive control. Representative Western Blots of three independent experiments are shown. Tubulin was used as a loading control. (**C**) Cell viability was measured following expression of RhoA, RhoC or GFP for 48 h. Metabolic activity was normalized to not induced cells. Data of three independent experiments were quantified and analyzed using two-way ANOVA. (**D**) BrdU incorporation after 48 h expression of RhoA, RhoC or GFP was measured to quantify the proliferation of MCF10A cells. DNA synthesis was normalized to the not induced cells. Data of three independent experiments were quantified and analyzed using two-way ANOVA. **p < 0.01, ***p < 0.001, ****p < 0.0001.

We intended to study the effect of RhoA/C expression or activation on the proliferation of breast epithelial cells. Therefore, we used sublines of human benign MCF10A cells, in which expression of either GFP, simultaneous expression of GFP and RhoA or expression of GFP and RhoC can be induced by addition of doxycycline. Time- and dose-dependent expression of the proteins following addition of doxycycline (+ dox) was analyzed by Western Blotting previously^8^. In a first set of experiments, colony formation assays were performed by growing the cell-lines in the absence or presence of doxycycline for 6 days. Expression of GFP had no effect on colony formation. In contrast, we detected a severe inhibition of the colony formation capacity of MCF10A cells following expression of GFP and RhoA or expression of GFP and RhoC, respectively (Fig. 1A). Colony formation depends on proliferation and viability as well as on differences of the cell size, contact inhibition and other cellular properties. First, we studied apoptosis. As expected, expression of RhoA or RhoC did not induce cell death. Staurosporine was used as positive control (Fig. 1B). Measurements of the metabolic activity as an indicator for cell viability showed a slight reduction to 90 or 80% in consequence of RhoA or RhoC overexpression. This moderate effect indicates that the reduced colony forming capacity of Rho expressing cells was not exclusively based on reduced viability (Fig. 1C). To measure proliferation, BrdU incorporation into newly synthesized DNA was detected. Doxycycline-induced expression of RhoA or RhoC reduced cell proliferation to about 50 to 60% compared to non-induced cells (Fig. 1D). In all experiments, RhoC had stronger effects than RhoA (compare Fig. 1B middle and right).

**Figure 2:**
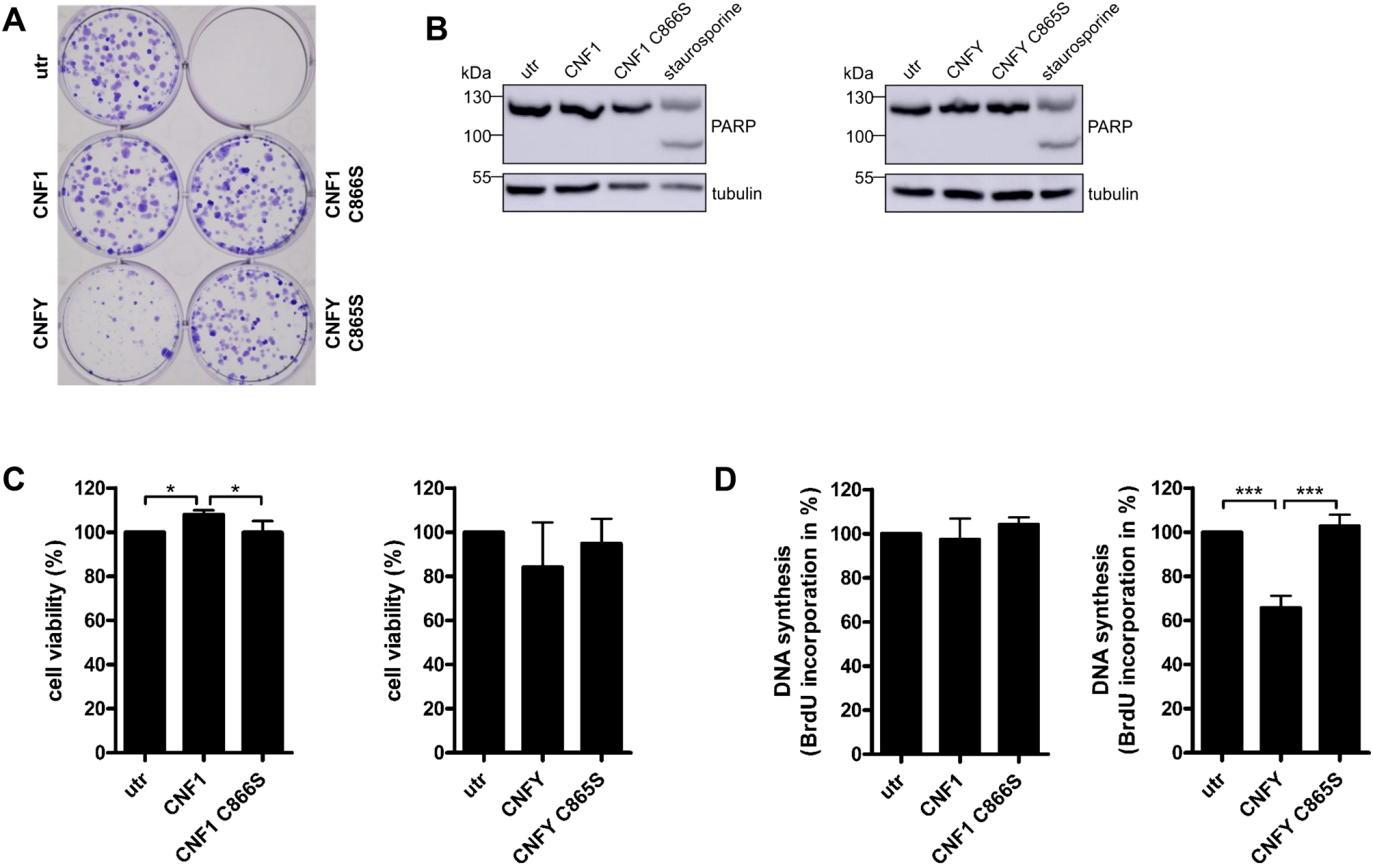
Intoxication with CNFY inhibits proliferation of MCF10A cells. (**A**) MCF10A wild-type cells were treated for 6 days with CNF1 or CNFY. The inactive toxin mutants served as a negative control. Cell colonies were stained with crystal violet (representative of n = 3). (**B**) Detection of PARP cleavage after intoxication with CNF1 or CNFY for 48 h. Treatment of the cells with 1 µM staurosporine for 16 h was used as a positive control, CNF1 C866S and CNFY C865S as negative controls, respectively. Representative Western Blots of three independent experiments are shown. Tubulin served as a loading control. (**C**) Cell viability of MCF10A wild-type cells was measured after CNF intoxication for 48 h. Metabolic activity was normalized to the untreated cells. Data of three independent experiments were quantified and analyzed using one-way ANOVA. (**D**) BrdU incorporation after intoxication with CNF1 or CNFY for 48 h. DNA synthesis was normalized to the untreated cells. Data of three independent experiments were quantified and analyzed using one-way ANOVA. *p < 0.05, ***p < 0.001.

To study whether the reduced metabolic activity and proliferation was based on the strong protein expression per se, we stimulated the endogenous pool of Rho GTPases by treatment of MCF10A cells with two bacterial toxins: CNFY predominantly activates RhoA,B,C whereas CNF1 activates Rac1, Cdc42 and RhoA,B,C. As controls, we used the respective catalytically inactive mutants of the toxins (CNF1 C866S and CNFY C865S). Effective uptake of the toxins into MCF10A cells and Rho activation was shown previously^8^. The colony formation assay was performed with MCF10A cells in the presence or absence of CNFs for 6 days. As shown in Fig. 2, a similar inhibitory effect on colony formation (A), metabolic activity (C) and proliferation (D) was achieved by activation of Rho GTPases due to treatment of the cells with CNFY. However, treatment with CNF1 had no effect on colony formation, indicating that activation of other Rho GTPases like Rac and/or Cdc42 may counteract the RhoA,B,C-induced inhibition of proliferation/colony formation^20^. To exclude an effect of the toxins on cell death, we additionally analyzed PARP-cleavage. Both toxins did not induce apoptosis of MCF10A cells (Fig. 2B). Metabolic activity was even slightly increased in the presence of CNF1 (Fig. 2C). To measure proliferation exclusively, BrdU incorporation was analyzed in the presence of the toxins or their inactive mutants, respectively. CNFY but not CNF1 reduced cell proliferation to about 60% compared to untreated controls (Fig. 2D).

### Rho-dependent expression of GPRC5A in MCF10A cell lines

**Figure 3:**
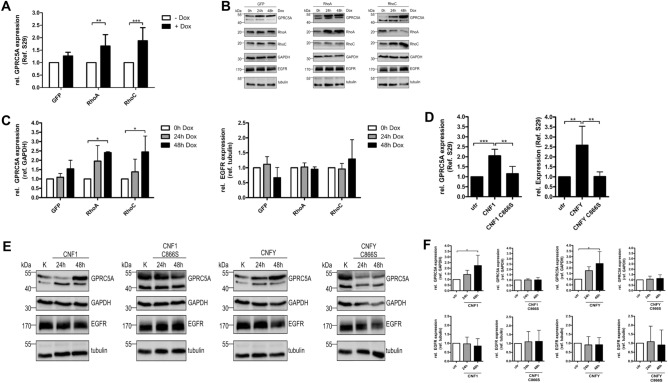
RhoA and RhoC induce the expression of GPRC5A. (**A**) GPRC5A mRNA level was measured by qRT-PCR following expression of RhoA, RhoC or GFP in MCF-10Atet cells (+ Dox) for 24 h. S29 was used as a housekeeping gene control. RNA levels after expression induction (+ Dox) were normalized to the untreated control (− Dox). Data of five independent experiments were quantified and analyzed using two-way ANOVA. (**B**) Representative Western Blot showing GPRC5A and EGFR protein levels following stimulation of RhoA, RhoC and GFP expressing MCF-10Atet cells for 0, 24 or 48 h with doxycycline (Dox). GAPDH and tubulin served as loading controls, respectively. Note that high expression of RhoC leads to decreased expression of RhoA, which has been described earlier^8^. (**C**) Quantification of B. GPRC5A protein level was normalized to GAPDH (left), EGFR protein level was normalized to tubulin (right). Data of three independent experiments were quantified and analyzed using two-way ANOVA. (**D**) GPRC5A mRNA level was measured by qRT-PCR after intoxication of MCF10A wild-type cells for 24 h with CNF1, CNF1 C866S, CNFY or CNFY C865S, respectively. S29 was used as a housekeeping gene control. RNA levels were normalized to the untreated (utr) control. Data of five independent experiments were quantified and analyzed using one-way-ANOVA. (**E**) Representative Western Blot (n = 3) showing GPRC5A and EGFR protein levels after intoxication with CNF1, CNF1 C866S, CNFY or CNFY C865S for 0, 24 or 48 h. GAPDH and tubulin served as loading controls. (**F**) Quantification of E. GPRC5A protein level was normalized to GAPDH (top), EGFR protein level was normalized to tubulin (bottom). The treatment with CNF1 and CNFY for two days GPRC5A expression was increased but the EGFR level was not affected. Data of three independent experiments were quantified and analyzed using one-way ANOVA. *p < 0.05, **p < 0.01, ***p < 0.001.

Recently, we performed a genetic screen to analyze genes regulated by expression of Rho proteins in MCF10A cells. We detected several pro-migratory genes upregulated following RhoA and/or RhoC expression^8^. Additionally, one of the genes with higher expression was RAI3^8^. It encodes for an orphan G-protein coupled receptor GPRC5A differently expressed in several human cancer entities. Interestingly, RAI3 was recently identified as a protein with a significant influence on proliferation of EGFR expressing cells^16^. Therefore, we asked whether upregulation of RAI3 might be involved in the Rho-dependent inhibition of proliferation of MCF10A cells. First, we validated the Rho-dependent induction of RAI3 mRNA levels by qRT-PCR and additionally studied the respective GPRC5A protein levels by Western Blotting. In line with the genetic screen, the amount of RAI3 mRNA increased about two-fold following induction of RhoA or RhoC expression by doxycycline for 24 h (Fig. 3A). Consistently, GPRC5A expression correlates with mRNA synthesis. It increased following induction of RhoA/C expression, whereas the level of EGFR did not change (Fig. 3B, quantification in Fig. 3C). We additionally studied the effect of Rho activation by toxin treatment and detected the same increase of RAI3 mRNA and GPRC5A protein in cells treated with CNF1 or CNFY, respectively (Fig. 3D,E, quantification in Fig. 3F). As expected, treatment of the cells with catalytically inactive toxin mutants had no effect. The data show that GPRC5A expression is upregulated downstream of RhoA and RhoC.

### Effect of GPRC5A expression on ligand-induced EGFR phosphorylation

**Figure 4:**
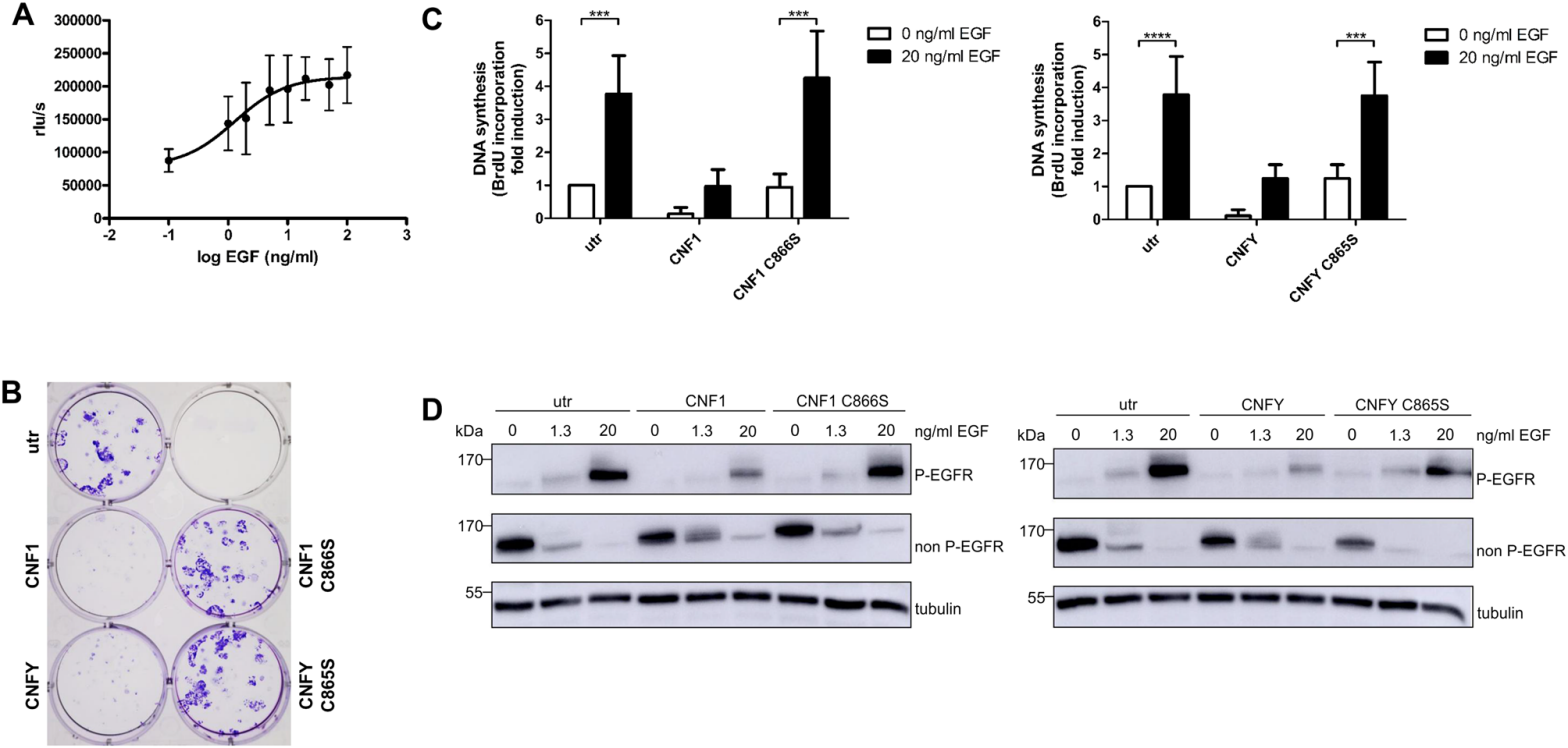
CNF1 and CNFY inhibit the EGF-dependent proliferation of MCF10A cells. (**A**) Dose versus response curve of BrdU incorporation after EGF stimulation. MCF10A cells were serum starved for 24 h and then stimulated with different EGF concentrations (0, 1, 2, 5, 10, 20, 50, 100 ng/ml) for 24 h. BrdU incorporation was measured as relative light units per seconds (rlu/s) and the EGF response was calculated using non-linear regression analysis. Data of three independent experiments are shown. EC_50_ = 1.3 ng/ml. Saturation of the stimulation was reached at 20 ng/ml EGF. (**B**) MCF10A cells were intoxicated with CNF1, CNFY or their inactive mutants for 2 days. After serum starvation for three h the cells were stimulated with 20 ng/ml EGF in medium containing 1% serum for eight days. Then, the colonies were fixed and stained with crystal violet (representative of n = 3). (**C**) MCF10A cells were treated with CNF1, CNFY or their inactive mutants for 48 h under serum starvation and were then stimulated with 0 or 20 ng/ml EGF for 24 h in the presence of the toxins, respectively. BrdU incorporation was measured and normalized as fold induction of the non-intoxicated unstimulated control. Data of three independent experiments were quantified and analyzed using two-way ANOVA. ***p < 0.001, ****p < 0.0001. (**D**) Western Blot analysis of EGFR phosphorylation after CNF intoxication. MCF10A cells were treated with CNF1, CNFY or their inactive mutants for 48 h under serum starvation. Then, the cells were stimulated with 0, 1.3 or 20 ng/ml EGF for five min. Representative Western Blots of three independent experiments are shown. Tubulin was used as a loading control.

In former studies, an inhibition of EGFR signaling by direct interaction with GPRC5A was shown^10^. To analyze the effect of Rho activation solely on EGF-dependent proliferation, we studied colony formation and DNA synthesis using serum starved MCF10A cells. As revealed by dose response analysis of BrdU incorporation into newly formed DNA, the optimal EGF concentration necessary to maximally stimulate proliferation of serum starved MCF10A cells is 20 ng/ml (EC_50_ = 1.3 ng/ml, Fig. 4A). Therefore, colony formation assays were performed with 20 ng/ml EGF in the presence or absence of the bacterial toxins or their catalytically inactive mutants, as indicated in Fig. 4B. In contrast to the experiments in full medium (containing 5% serum, Fig. 1A), colony formation was blocked in medium with low serum (1%, supplemented with EGF) in the presence of CNF1 or CNFY, respectively. In line with the colony formation assay, both toxins reduced basal and EGF-stimulated BrdU incorporation, whereas the catalytically inactive mutants had no effect (Fig. 4C). These data indicate that Rho activation blocked EGF-dependent proliferation. Therefore, we studied direct phosphorylation of the EGFR following EGF stimulation in the presence and absence of the toxins by Western Blotting. For detection of EGFR phosphorylation, we used an antibody against phospho-EGFR (Fig. 4D, top lane) and a second antibody, which detects only the non-phosphorylated EGFR (Fig. 4D, middle lane). EGF-stimulated phosphorylation was reduced by treatment with the toxins. Rho stimulation by CNFs led to reduced EGF-dependent receptor phosphorylation and proliferation probably by enhanced expression of GPRC5A. CNF1 and CNFY led to reduced basal DNA synthesis and impaired the EGF-dependent proliferation, respectively.

### Expression of GPRC5A is sufficient to inhibit proliferation

To analyze, whether expression of GPRC5A is sufficient to influence EGFR signaling, we transiently expressed the hepta-helical receptor in MCF10A cells by viral transduction. Following addition of virus-containing supernatants (empty vector control and GPRC5A, respectively), cells were serum starved for 2 days, stimulated with EGF (1.3 and 20 ng/ml, respectively) for 5 min. Cleared lysates were analyzed for expression of GPRC5A, phospho-EGFR and total EGFR by Western Blotting.

**Figure 5:**
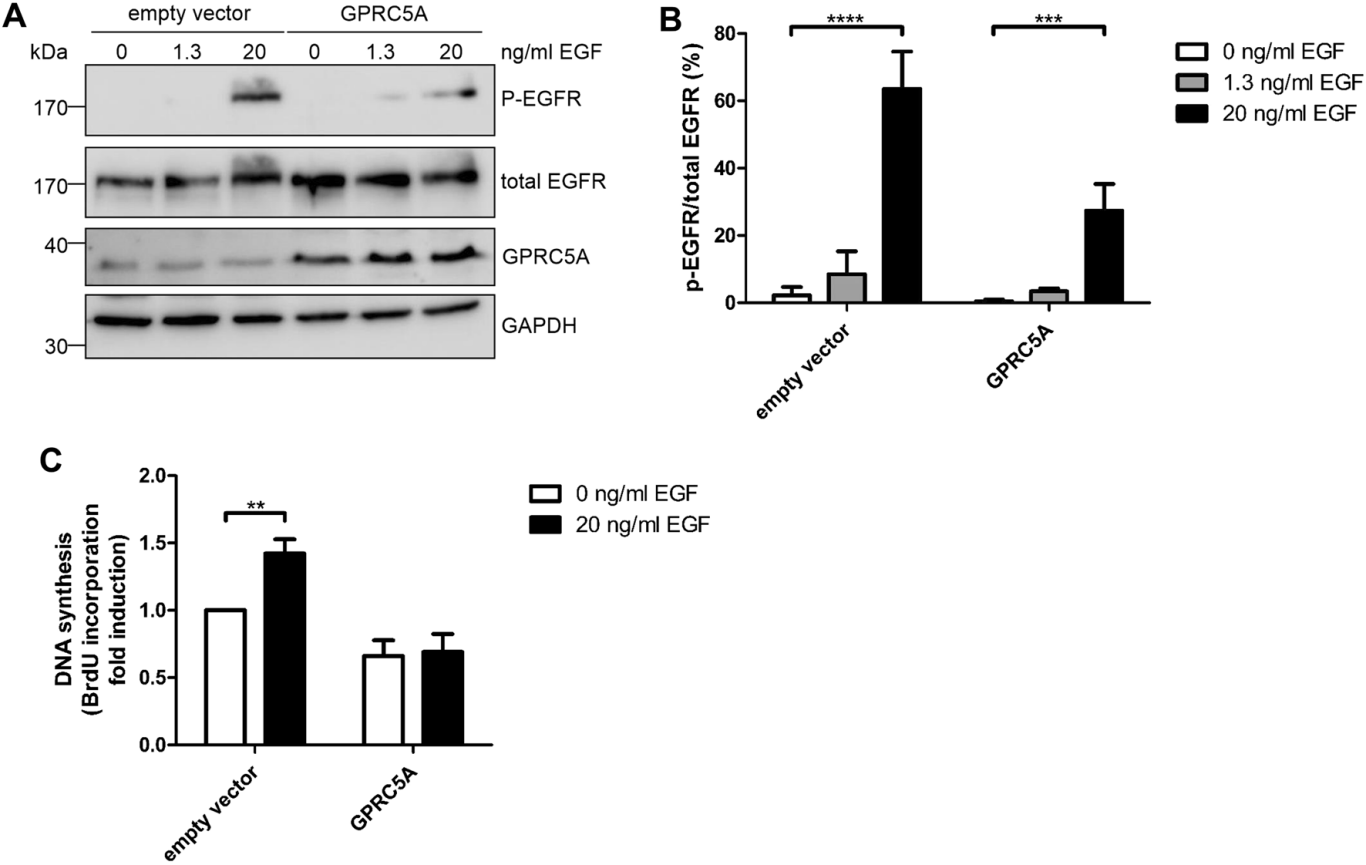
Expression of GPRC5A reduces EGF stimulated phosphorylation of EGFR. (**A**) Western Blot analysis of EGFR phosphorylation following transient expression of GPRC5A. In MCF10A cells, RAI3 expression was induced by viral transduction using pMiBerry-GPRC5A or empty vector as control. After serum starvation for 48 h, the cells were stimulated with 0, 1.3 or 20 ng/ml EGF for five min. Representative Western Blots of three independent experiments are shown. GAPDH was used as loading control. (**B**) Quantification of A. Data of three independent experiments were quantified and analyzed using two-way ANOVA. ***p < 0.001, ****p < 0.0001. (**C**) In MCF10A cells GPRC5A was expressed by viral transduction. After serum starvation for 48 h, the cells were stimulated with 20 ng/ml EGF and incubated with BrdU for 4 h. DNA synthesis was normalized to the unstimulated empty vector control. Data of three independent experiments were quantified and analyzed using two-way ANOVA. **p < 0.01.

As shown in Fig. 5A, the cells show about 2 to 3-fold higher expression compared to the empty vector-transduced MCF10A cells. Phosphorylation of the EGFR following stimulation with EGF was significantly reduced (by about 50%) in GPRC5A expressing cells (Fig. 5B). Additionally, to measure proliferation, BrdU incorporation into newly synthesized DNA was detected in GPRC5A overexpressing cells. Therefore, transduced cells (empty vector control and GPRC5A, respectively) were seeded into 96 well plates, serum starved for two days and stimulated with EGF (20 ng/ml) for 4 h in the presence of BrdU. As shown in Fig. 5C, EGF-stimulated proliferation of the empty vector transduced cells was increased about 1.5 times compared to the unstimulated cells (set to 1). In contrast, there was no increased BrdU incorporation detectable in GPRC5A expressing cells following exposure to EGF. The data show that EGF-stimulated proliferation was inhibited due to enhanced expression of GPRC5A.

### GPRC5A is required for stabilization of monomeric EGFR

**Figure 6:**
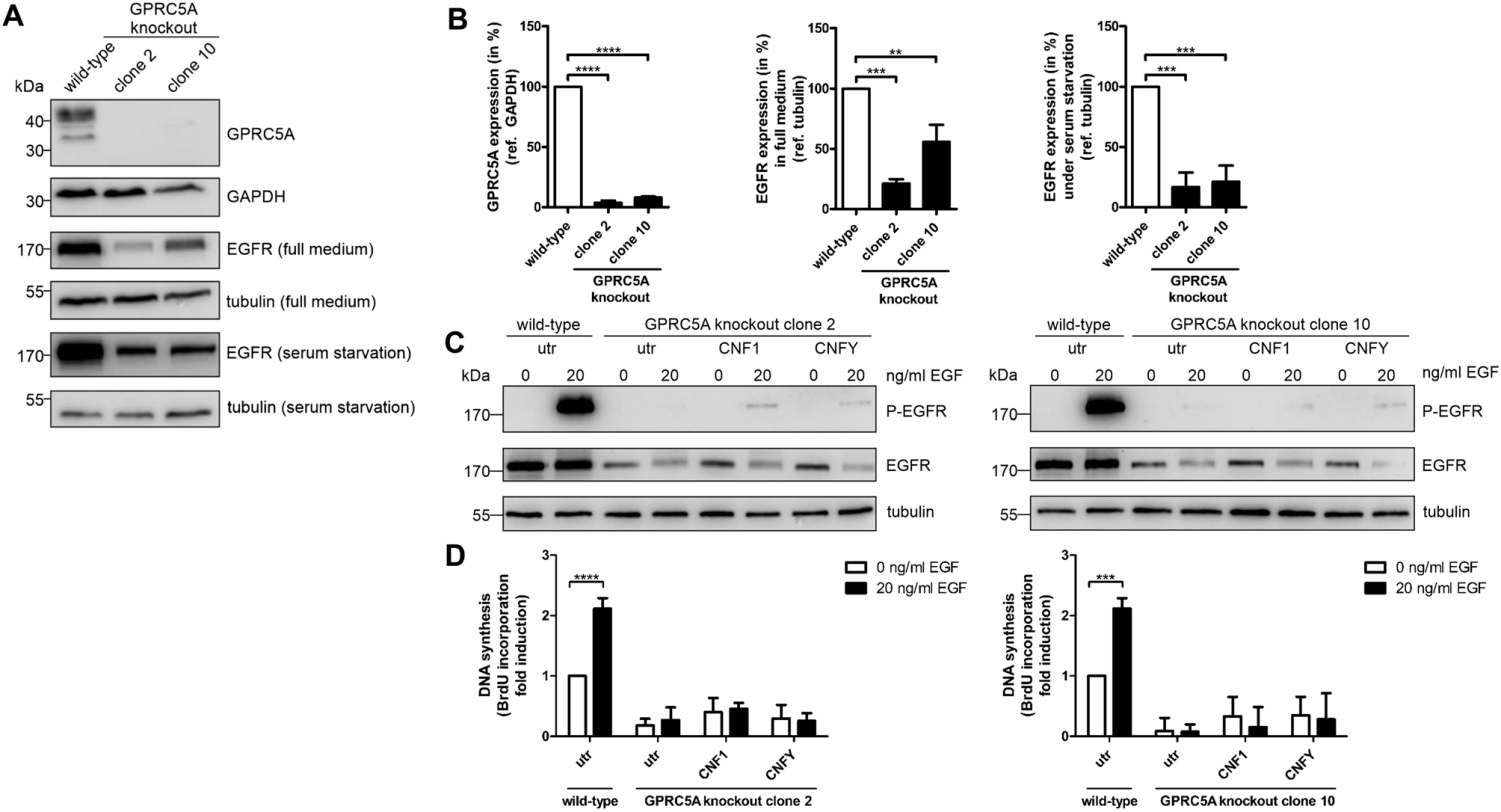
Knockout of GPRC5A in MCF10A cells inhibits EGF-dependent proliferation due to downregulation of EGFR. (**A**) Western Blot analysis of MCF10A wild-type and knockout cells in full medium and under serum starvation, respectively. GAPDH and tubulin were used as a loading controls. Representative Western Blot of three independent experiments are shown. (**B**) Quantification of A. GPRC5A expression was normalized to GAPDH, EGFR expression to tubulin. Data of three independent experiments were analyzed using one-way ANOVA. **p < 0.01, ***p < 0.001, ****p < 0.0001. (**C**) Western Blot analysis of EGFR phosphorylation after CNF intoxication of MCF10A GPRC5A knockout cells. The cells were treated with CNF1 or CNFY for 48 h under serum starvation. MCF10A wild-type cells were grown equally without intoxication as control. Then, the cells were stimulated with 0 or 20 ng/ml EGF for five min. Representative Western Blots of three independent experiments are shown. Tubulin was used as a loading control. (**D**) MCF10A wild-type and GPRC5A knockout cells were grown for 48 h under serum starvation and were then stimulated with 0 or 20 ng/ml EGF for 24 h. The knockout cells were furthermore intoxicated with CNF 1 or CNFY, respectively. BrdU incorporation was measured and normalized as fold induction of the non-intoxicated unstimulated MCF10A wild-type cell control. Data of three independent experiments were quantified and analyzed using two-way ANOVA. ***p < 0.001, ****p < 0.0001.

To study the effect of Rho activation per se on EGFR signaling and proliferation, we performed a knockout of GPRC5A in MCF10A cells proven by Western Blot (Fig. 6A, quantification in Fig. 6B). Interestingly, the expression of EGFR decreased to 20 and 55% and under serum starvation to 15 and 20%, respectively.

To analyze the effect of Rho activation on EGF-dependent proliferation, we studied EGFR phosphorylation and DNA synthesis using serum starved MCF10A cells as described above and stimulated them with 20 ng/ml EGF. In GPRC5A knockout cells, the amount of phosphorylated EGFR was significantly diminished and additional activation of Rho GTPases by CNF1 or CNFY had no effect (Fig. 6C). BrdU incorporation in RAI3 depleted cells was significantly reduced under serum starvation and almost blocked, even after EGF stimulation. As expected, intoxication with CNF1 or CNFY was not sufficient to reactivate proliferation (Fig. 6D). Our data show that the amount of EGFR is influenced by the expression of GPRC5A and not mediated by toxin-induced Rho activation. The monomer seems to be stabilized in the presence of GPRC5A, which on the one hand inhibits degradation and on the other hand negatively influences dimerization and signaling of the receptor.

### Rho activation in breast cancer cells carrying an activating Ras mutation had no effect on proliferation, whereas knockout of GPRC5A increased cell division

**Figure 7:**
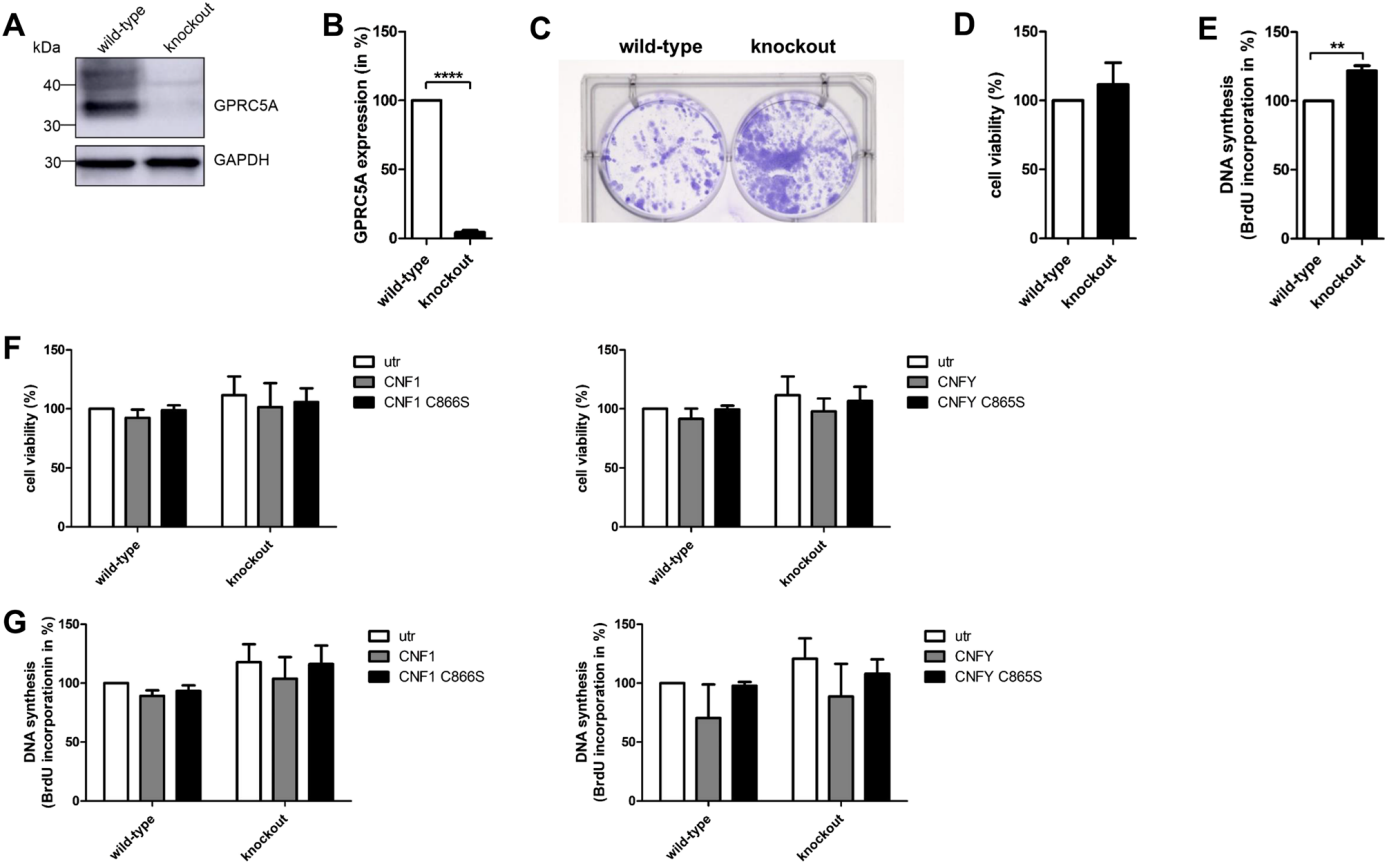
Knockout of GPRC5A in MDA-MB-231 cells does not inhibit proliferation. (**A**) Western Blot analysis of MDA-MB-231 wild-type and knockout cells. GAPDH was used as loading control (representative of n = 3). (**B**) Quantification of A. GPRC5A expression was normalized to GAPDH. Data of three independent experiments were analyzed using t-test. ****p < 0.0001. (**C**) MDA-MB-231 wild-type and knockout cells were grown for ten days. Cell colonies were stained with crystal violet (n = 3). (**D**) MDA-MB-231 wild-type and GPRC5A knockout cells were grown for 48 h. Cell viability was measured and normalized to the wild-type cells. Data of three independent experiments were quantified and analyzed using t-test. (**E**) MDA-MB-231 wild-type and GPRC5A knockout cells were grown for 48 h. BrdU incorporation was measured and normalized to the wild-type cells. Data of three independent experiments were quantified and analyzed using t-test. **p < 0.01. (**F**) Cell viability of MDA-MB-231 wild-type and GPRC5A knockout cells was measured after CNF intoxication for 48 h. Metabolic activity was normalized to the untreated wild-type cells. Data of three independent experiments were quantified and analyzed using two-way ANOVA. (**G**) BrdU incorporation of MDA-MB-231 wild-type and GPRC5A knockout cells was measured after CNF intoxication for 48 h. DNA synthesis was normalized to the untreated wild-type cells. Data of three independent experiments were quantified and analyzed using two-way ANOVA.

Our data suggest an inhibitory effect of GPRC5A on proliferation of breast epithelial cells most likely by diminished EGFR stimulation. To verify that this influence on proliferation was predominantly based on EGFR signaling, the effect of the toxins was analyzed on MDA-MB-231 breast cancer cells bearing an activating Ras mutation and are therefore independent on EGFR signaling. We induced a knockout of RAI3 in MDA-MB-231 cells by CRISPR-Cas9 and verified the functional gene knockout by Western Blot as shown in Fig. 7A (quantification in Fig. 7B). As expected, knockout of GPRC5A increased the colony forming capacity of MDA-MB-231-cells (Fig. 7C) and stimulated BrdU incorporation by about 20% compared to the wild-type cells but did not affect cell viability (Fig. 7D,E). The data indicate an anti-proliferative effect of GPRC5A also in cells with dominant active Ras. Our data are in line with recent experiments in which knockdown of GPRC5A in MDA-MB-231 breast cancer cells promoted colony formation and proliferation^16^. Neither cell viability nor proliferation of MDA-MB-231 wild-type and GPRC5A knockout cells was affected by CNF1 or CNFY most likely because EGFR downstream signaling was already activated in MDA-MB-231 cells (Fig. 7F,G). The data show that it is not the effect of the Rho-activating toxins which influences the proliferation and colony formation but Rho-induced expression of GPRC5A and inhibition of EGFR signaling.

## Discussion

**Figure 8:**
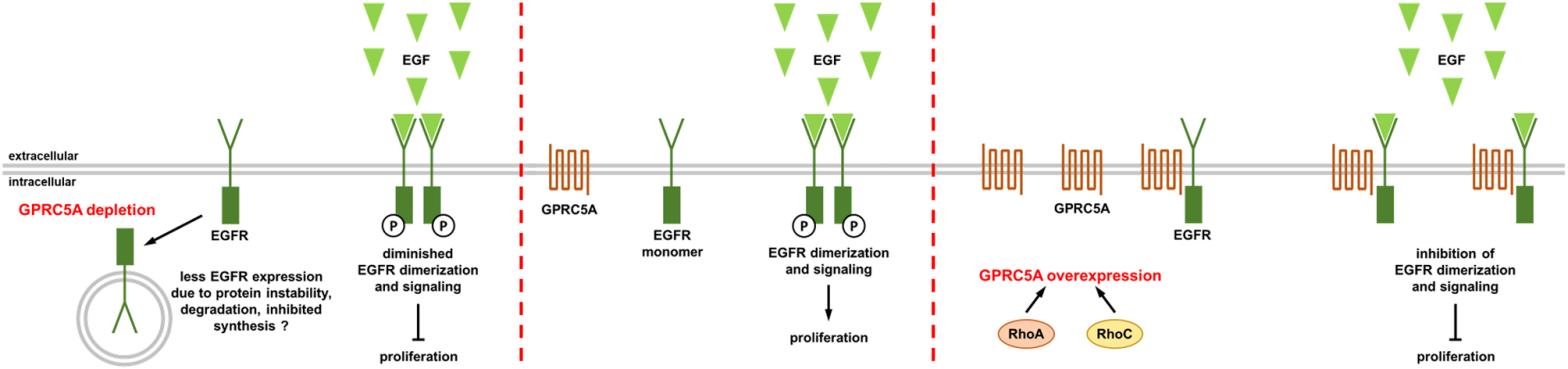
Model depicting various effects of GPRC5A on EGF-dependent proliferation. Under physiological GPRC5A expression it interacts with the unstimulated EGFR and stabilizes the protein at the cell membrane. After EGF stimulation the EGFR dissociates from RAI3 and forms dimers for activation of the kinase domain leading to further signal transduction (middle). When GPRC5A is overexpressed, it behaves like a dissociation inhibitor. Upon stimulation the EGFR monomers cannot form dimers. Less EGFR phosphorylation results in diminished proliferation (right). GPRC5A depletion results in downregulation of the EGFR and thereby inhibits proliferation. The mechanism has to be further investigated (left).

Cancer is an extremely heterogeneous disease and even cells of one cancer entity often show a wide variety of different gene profiles and morphological characteristics. The epidermal growth factor receptor (EGFR) plays a critical role in cancer since it mediates proliferation by activation of Ras and STAT. EGFR kinase inhibitors have successfully developed. Recently, it was shown that an orphan G protein coupled receptor (GPCR) interacts with EGFR, sequestering it as a monomer and thereby inhibiting receptor signaling. In line with this, expression of GPRC5A is low in non small cell lung cancer (NSCLC)^21^. Moreover, GPRC5A knockout mice developed spontaneous lung cancer^11^ and GPRC5A loss was associated with increased cell proliferation and resistance to cell death^22^. The gene was thus designated a tumor suppressor. In pancreatic cancer however, knockdown of RAI3 (the gene for GPRC5A) led to decreased proliferation and reduced migration, indicating a pro-metastatic role for GPRC5A in pancreatic cancer^14^. In breast cancer, the picture is diverse: According to the “bioportal” website, GPRC5A expression analysis revealed more breast tumor tissues with protein amplification than with deletions. However, the diverse role of GPRC5A in tumor formation is reflected by recent studies with breast cancer cell lines. Knockdown of GPRC5A promotes colony formation and proliferation by activation of EGFR in MDA-MB-231 cells but showed no effect in MCF7 cells expressing only low amounts of EGFR^16^. In cells carrying an activating Ras mutation, GPRC5A has less effect on proliferation and survival. This proves that the effect of GPRC5A on proliferation is mediated by its influence on EGFR, which also activates other signaling pathways as for example PI3K. Our data indicate that only the EGFR dimer is stably expressed at the cell membrane, whereas the empty receptor needs GPRC5A to be stabilized, suggesting that the level of GPRC5A on the one hand interferes with dimerization and signaling of EGFR but on the other hand stabilizes the EGFR monomer against degradation (model depicted in Fig. 8).

The inhibitory effect of GPRC5A on proliferation may vary, if other EGFR family members are expressed in that sense that Her2 stabilizes EGFR. It is not known whether human EGF receptor 2 (Her2) also interacts with GPRC5A and whether its signaling is also blocked. In a recent publication by Fichter et al. it is shown that homo- and heterodimers of EGFR and Her2 form differently in diverse tissues, which may explain varying effects of GPRC5A expression^23^.

Here, we identified RAI3 as a gene upregulated by Rho GTPase signaling in breast epithelial cells. Expression of GPRC5A significantly reduced proliferation of the cells. Moreover, knockout of RAI3 also inhibited EGF-dependent proliferation due to EGFR downregulation. The GPCR is an orphan receptor. Neither the ligand nor its intracellular signaling partner is known. However, recent deletion studies showed that it is not the signaling of the GPCR to heterotrimeric G-proteins, which influences EGFR activity. Rather the transmembrane part of GPRC5A seems to be important because deletion of the N-terminus, or deletion of the C-terminus of the hepta-helical receptor did not affect its inhibitory action on EGFR signaling^10^.
